# Point-of-care characterization and risk-based management of oral lesions in primary dental clinics: A simulation model

**DOI:** 10.1371/journal.pone.0244446

**Published:** 2020-12-31

**Authors:** Stella K. Kang, Rahul D. Mali, R. Scott Braithwaite, Alexander R. Kerr, John McDevitt

**Affiliations:** 1 Department of Radiology, New York University Grossman School of Medicine, New York, New York, United States of America; 2 Department of Population Health, New York University Grossman School of Medicine, New York, New York, United States of America; 3 Department of Medicine, New York University Grossman School of Medicine, New York, New York, United States of America; 4 Department of Oral and Maxillofacial Pathology, Radiology, and Medicine, New York University College of Dentistry, New York, New York, United States of America; 5 Department of Biomaterials, New York University College of Dentistry, New York, New York, United States of America; 6 Chemical and Biomolecular Engineering, Tandon School of Engineering, New York University, Brooklyn, New York, United States of America; University of Wisconsin, UNITED STATES

## Abstract

**Objectives:**

Oral potentially malignant disorders (OPMDs) encompass histologically benign, dysplastic, and cancerous lesions that are often indistinguishable by appearance and inconsistently managed. We assessed the potential impact of test-and-treat pathways enabled by a point-of-care test for OPMD characterization.

**Materials and methods:**

We constructed a decision-analytic model to compare life expectancy of test-treat strategies for 60-year-old patients with OPMDs in the primary dental setting, based on a trial for a point-of-care cytopathology tool (POCOCT). Eight strategies of OPMD detection and evaluation were compared, involving deferred evaluation (no further characterization), prompt OPMD characterization using POCOCT measurements, or the commonly recommended usual care strategy of routine referral for scalpel biopsy. POCOCT pathways differed in threshold for additional intervention, including surgery for any dysplasia or malignancy, or for only moderate or severe dysplasia or cancer. Strategies with initial referral for biopsy also reflected varied treatment thresholds in current practice between surgery and surveillance of mild dysplasia. Sensitivity analysis was performed to assess the impact of variation in parameter values on model results.

**Results:**

Requisite referral for scalpel biopsy offered the highest life expectancy of 20.92 life-years compared with deferred evaluation (+0.30 life-years), though this outcome was driven by baseline assumptions of limited patient adherence to surveillance using POCOCT. POCOCT characterization and surveillance offered only 0.02 life-years less than the most biopsy-intensive strategy, while resulting in 27% fewer biopsies. When the probability of adherence to surveillance and confirmatory biopsy was ≥ 0.88, or when metastasis rates were lower than reported, POCOCT characterization extended life-years (+0.04 life-years) than prompt specialist referral.

**Conclusion:**

Risk-based OPMD management through point-of-care cytology may offer a reasonable alternative to routine referral for specialist evaluation and scalpel biopsy, with far fewer biopsies. In patients who adhere to surveillance protocols, POCOCT surveillance may extend life expectancy beyond biopsy and follow up visual-tactile inspection.

## Introduction

Worldwide, there are over 500,000 new cases of oral cavity and pharyngeal cancer diagnosed each year [1]. In the United States, more than 53,000 new cases and nearly 10,860 deaths were estimated in 2019, constituting approximately 4% of all cancers in men and 2% in women [2]. More than 90% of oral cavity cancers are squamous cell carcinomas (OSCC), which arise in the oral mucosa [3, 4]. Despite advances in treatment, the long-term prognosis for patients with OSCC remains poor with a five-year survival rate of approximately 64% [5, 6]. This poor prognosis is largely attributed to diagnostic delays as the survival rate increases dramatically to 83% when OSCC is detected at an early stage. Unfortunately, in the United States, only one-third of OSCC cases are detected as localized disease, and therefore the majority lose the opportunity to undergo less radical and chemotoxic therapies [7, 8]. Outcomes of OSCC may be improved through earlier detection and diagnosis of oral cancer and oral potentially malignant disorders (OPMDs). OPMDs, including leukoplakia and erythroplakia, are epithelial lesions detected by a visual and tactile examination which do not have the appearance of clearly benign conditions, and therefore may require further investigation (i.e. tissue biopsy) to assess for epithelial dysplasia or squamous cell carcinoma.

The American Dental Association recommends screening for OPMDs as a part of routine dental care in order to improve the chances of early intervention for cancerous lesions. Still, examination and management remain inconsistent in dental care, where most OPMDs are typically encountered.[9, 10] Some reluctance to screen is attributable to the lack of evidence that the small yield of cancers in populations with low cancer prevalence would outweigh the large numbers of potential false positive findings on visual and tactile exam [11]. Currently, it is estimated that 3% of patients who undergo a visual and tactile examination in a dental office receive a clinical diagnosis of a OPMD, and among these patients approximately 5% will contain OSCC [12]. Furthermore, common and readily discoverable clinical risk factors such as older age, tobacco and alcohol use increase risk at least 30-fold from baseline [13–15]. With an effective chairside diagnostic test, the projected annual diagnostic yield could fall within the range previously accepted for other cancers, approximately 1 invasive cancer per 1,000 screened patients [16–18]. Thus, current major barriers to improved control of OSCC are the limited sensitivity and specificity of the visual and tactile exam to identify “at risk” lesions (i.e. cancer and precancerous lesions with propensity for subsequent cancer development), while minimizing referrals for specialist visits and biopsies for mostly benign lesions.

A novel point-of-care cytological test called the Point-of-care Oral Cytopathology Tool (POCOCT) offers precision diagnostic information to guide lesion management and mitigate the potential harms of both under- or over-testing. This computer-vision based technology rapidly scans and categorizes cells collected by mucosal brushing, and combines these features with other clinical risk predictors to determine the likelihood of dysplasia or malignancy. In a diagnostic performance trial involving 714 prospectively recruited patients with OPMDs with matched histopathologic diagnoses, lesion categories were evaluated across the spectrum of disease from benign to dysplasia to carcinoma;[19] POCOCT performance was moderate to excellent with area-under-the-curve ranging from 0.84–0.88 for separation at selected points along the spectrum from benign to dysplastic to carcinoma. A numeric index was developed and validated using cytology data from the trial, resulting in a weighted and aggregated continuous score comprising four diagnostic cut points. Specifically, the classifier provided a single, continuous severity score comparable to performance of expert pathologists (with individual class prediction accuracy from 76.0% to 97.6%), and therefore POCOCT does not require a cytopathologist for interpretation of results. We constructed a decision-analytic model based on the results of the trial to evaluate the potential of risk-based management of OPMDs in the primary dental setting, by comparing deferral of evaluation (no initial referral) with risk-tailored management using POCOCT, and also the currently recommended strategy of prompt referral of all patients for specialist evaluation and scalpel biopsy.

## Materials and methods

### Decision analytic model summary

**Fig 1:**
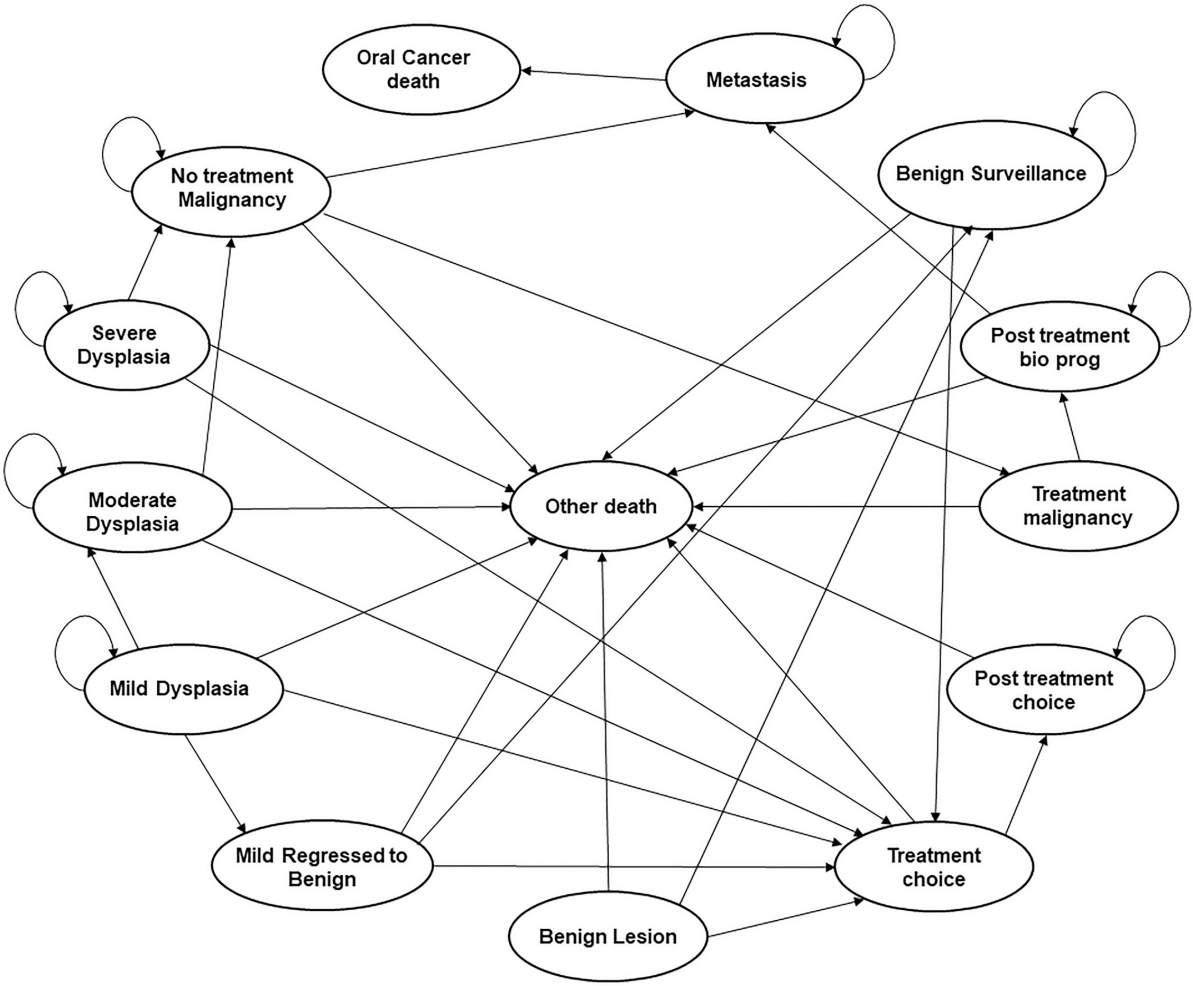
State transition diagram for the strategies using the POCOCT. Mild dysplasia may be surgically removed in pathways designated for treatment, or if monitored, regress to benign lesions or progress to moderate dysplasia. Similarly, moderate and severe dysplasia are treated if in a designated treatment pathway, or if monitored, can progress to malignancy. Malignant lesions are treated if detected, or if left untreated may metastasize. Represented causes of death include metastatic disease or other all-cause mortality.

A state-transition model was developed using TreeAge Pro version 2019 (TreeAge Software, Inc., Williamstown, MA, USA). A state-transition (or Markov) model is a type of mathematical model for simulating patients through a sequence of particular health states [20]. Our model simulated a hypothetical cohort of 60-year-old men with asymptomatic OPMDs until death, using a cycle length of 1 month. The model incorporated different risks of disease progression and different chances of treatment success for patients presenting with the various types of OPMDs including benign, dysplastic, and malignant lesions. Our primary outcome was Life Expectancy (LE), which would be expected to reflect the benefit (cancers avoided or successfully treated) as well as harm (unnecessary biopsies) tradeoffs inherent in screening-related decisions. The patients simulated in the model progress by increasing grade of dysplasia or stage of cancer, can be subjected to treatments, and die due to OSCC or all-cause mortality (**Fig 1**).

The aim of opportunistic screening for oral cancer/OPMDs is to have a high sensitivity for detecting significant disease (e.g. early cancers or severely dysplastic lesions). Having stated that, mildly or moderately dysplastic OPMDs can progress and transform. As such, the threshold/cut-off that we set for POCOCT can have trade-offs. A high threshold results in treatment of only higher grade disease and monitoring lower grade disease that could transform to cancer. A low threshold of treatment risks over-referral for unnecessary specialist evaluation/biopsies and possibly minimal improvement in cancer-specific outcomes. The overall performance of POCOCT for each histopathologic cut-off (or split) was generated from a prior trial [19]. As such, we chose to analyze outcomes of each diagnostic cut-off.

### Diagnostic strategies

**Fig 2:**
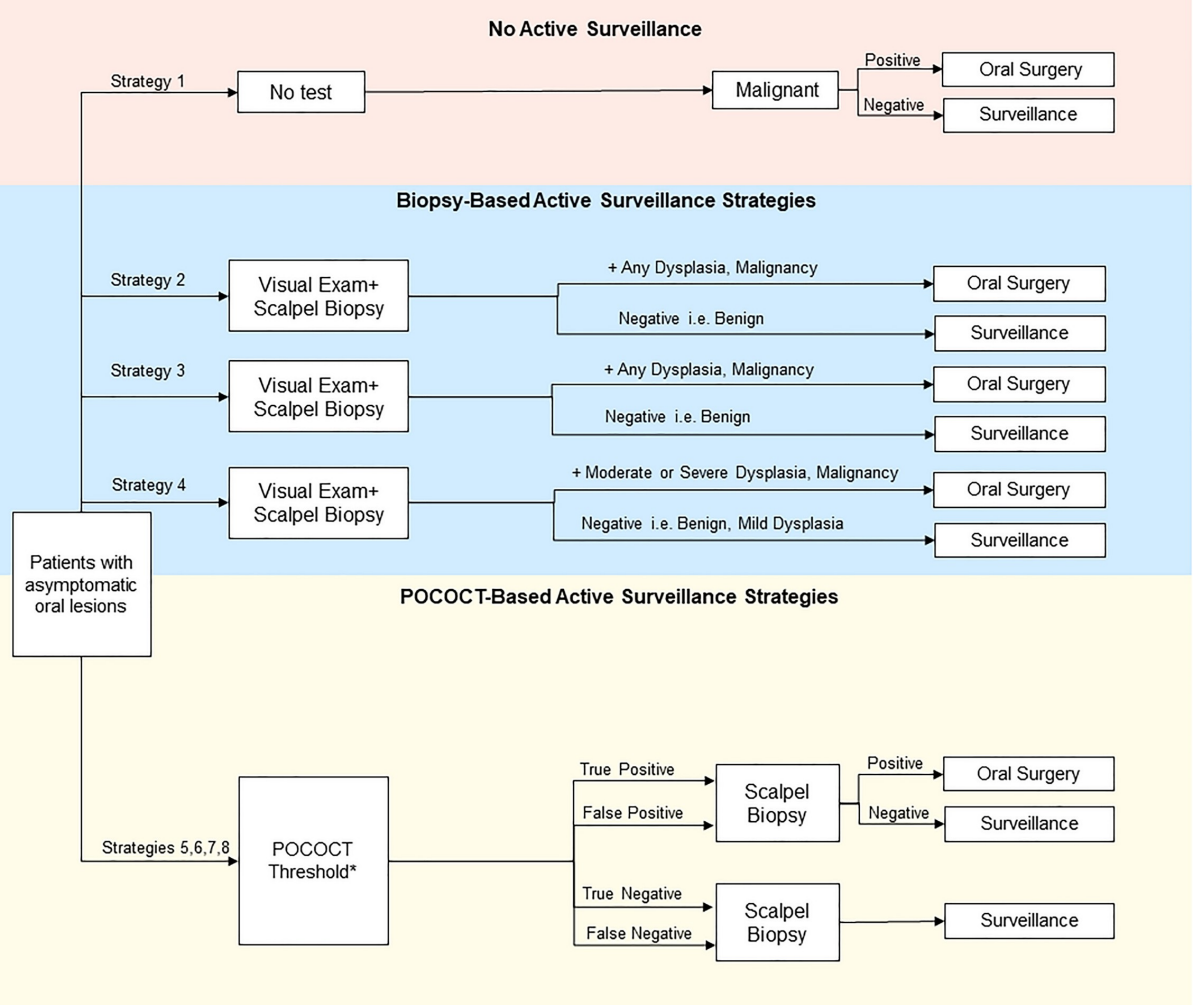
Simplified schematic of decision-analytic model summarizes active management strategies and testing consequences in the management of OPMDs. In addition to these management strategies, an additional “deferred evaluation and testing” pathway was constructed for comparison. *POCOCT generates a numerical score using the four different diagnostic splits among the 6 POCOCT risk categories spanning benign, dysplastic, and carcinomatous results. These diagnostic splits (e.g. mild|moderate dysplasia) were tested to determine the ideal thresholds for performing confirmatory biopsy and treating dysplasia or malignancy. For instance, use of the mild|moderate split entailed biopsy if positive, then surgical resection of moderate or severe dysplasia and carcinoma, while benign lesions and mild dysplasia histologic findings were placed on POCOCT-based surveillance. Similarly, splits of benign|mild, moderate|severe, and low-risk|high-risk moderate dysplasia were applied to separate test-treat pathways.

Using each diagnostic split to create multiple possible test-treat pathways for OPMDs, we compared life expectancies using the model. This assessment included varied thresholds for further evaluation or treatment based upon the different diagnostic evaluations. In the standard care test-and treat pathways the decision to treat is based on the various histopathological diagnoses rendered by specialist biopsy. In the non-invasive pathways, the decision to refer and treat is based on a positive POCOCT test outcome and given that the threshold or cut-off for a positive POCOCT test is an accurate surrogate for histopathology, it is possible to set these test thresholds across multiple diagnostic splits. The sensitivity and specificity of POCOCT for each diagnostic split was defined in a prior diagnostic accuracy trial [16, 18]. The splits include benign vs any dysplasia/carcinoma (benign|mild split) where, as an example, OPMDs that have no actual dysplasia and are benign would receive a negative POCOCT outcome and OPMDs harboring mild dysplasia or worse histopathology would receive a positive POCOCT outcome, benign/mild dysplasia vs moderate or higher grade dysplasia/carcinoma (mild|moderate split), benign/mild/moderate dysplasia vs severe dysplasia/carcinoma (moderate|severe split), and finally, a low vs high risk dysplasia which is based on the new WHO dichotomous classification system (benign, mild, lower end of moderate dysplasia vs higher end of moderate dysplasia, severe dysplasia/carcinoma (low|high split) (**Fig 2**). These POCOCT pathways were compared against alternative strategies: prompt referral for specialist evaluation and biopsy of all OPMDs visually detected by primary care dentists (i.e. representing the recommended practice pattern), or deferral of further evaluation of OPMDs by a specialist unless frankly malignant (i.e. representing a less common practice pattern) [9]. Strategies based on specialist consultation and biopsy entailed various histologic thresholds for surgical treatment, mirroring the POCOCT strategies.

The complete list of strategies included: (1) deferral of further evaluation of visible OPMDs by a specialist; (2) prompt referral for specialist evaluation and biopsy for OPMDs, surgery if biopsy is positive for any grade of dysplasia or carcinoma, and no option for surveillance; (3) prompt referral for specialist evaluation and biopsy for OPMDs, surgery if biopsy is positive for any grade of dysplasia or carcinoma, and surveillance for benign lesions; (4) prompt referral for specialist evaluation and biopsy for OPMDs, surgery if biopsy is positive for moderate or severe dysplasia or carcinoma, and surveillance for benign lesions and mild dysplasia; (5) POCOCT benign|mild dysplasia diagnostic split, with surgery for any dysplasia or carcinoma, and surveillance for benign lesions; (6) POCOCT mild|moderate dysplasia diagnostic split, with surgery for moderate or severe dysplasia or carcinoma, and surveillance for benign lesions and lesions with mild dysplasia; (7) POCOCT “low risk|high risk” diagnostic split, with surgery for “higher-risk” dysplasia (encompassing more severe degree of moderate dysplasia and severe dysplasia) or carcinoma, and surveillance for benign and “lower-risk” dysplasia; and (8) POCOCT moderate|severe dysplasia diagnostic split, with surgery for severe dysplasia and carcinoma, and surveillance for benign lesions or lesions with mild or moderate dysplasia.

To compare against strategies representing prompt screening and evaluation for OPMDs, one model pathway represented deferral of OPMD management and characterization and its consequences. In the deferral strategy, only symptomatic lesions were referred (i.e. with higher suspicion for frank malignancy and with higher proportion in late stage).

In the strategies with prompt referral for biopsy of OPMDs (strategies 2–4), the indications for surgery were varied according to the histopathology of the lesion. In one biopsy strategy (#2), the patients with benign results did not undergo visual surveillance, in order to model effects of possible sampling error with biopsy. In strategy 3, patients underwent surgery if biopsy was positive for any dysplasia or carcinoma and benign results warranted visual examinations every six months for the first year and thereafter annually up to five years. Likewise, other strategies involving visual exam-based surveillance of biopsy findings below the treatment threshold applied a schedule of every six months for the first year and thereafter annually up to 5 years. In the case of deferred oral cavity screening, patients with localized stage (stage 1 or 2) malignant lesions had 13% probability of presenting for a visual-tactile exam followed by scalpel biopsy and treatment [21, 22].

In the four strategies with OPMD management based on POCOCT results, we used the diagnostic risk categories to vary the treatment threshold, such that in the most conservative treatment approach OPMDs were referred for scalpel biopsy and treated if any degree of dysplasia or carcinoma was indicated by POCOCT (Strategy 5), and alternatively, only progressively higher thresholds for biopsy and treatment were pursued (Strategies 6–8). Surveillance in these point-of-care testing strategies entailed repeat POCOCT evaluation in 6 months, then at 1 year, and then yearly up to 5 years. In the model’s surveillance strategies, patients were subject to the accuracy of the follow up test (either POCOCT or visual-tactile exam) and then assumed to receive recommendation for scalpel biopsy for a positive exam.

### Model inputs

The model incorporated patient age, comorbidity status, misclassification of histology along the spectrum of dysplasia and malignancy, and risks of cancer progression. All-cause mortality rates were derived from U.S. life tables [23]. Comorbidity-related mortality risks were represented using the Charlson comorbidity score [24]. Transition probabilities for health states were derived from the literature and our calibration models (**Table 1**). The sensitivity and specificity of POCOCT for each diagnostic split was informed using diagnostic trial findings [19]. The baseline prevalence of benign lesions, dysplasia and carcinoma among OPMDs were based on the literature [25]. The probability of progression of late stage cancer to metastasis was calibrated using a separate disease model, incorporating the reported survival rates of patients with metastatic disease and survival data in patients presenting with late stage disease [2].

**Table 1:** Major parameters used in the model, including base case values and range for sensitivity analysis.

Description	Value	Low	High	Source
Initial proportion of OPMDs containing malignancy	0.05	0.02	0.075	[25, 26]
Initial proportion of OPMDs containing mild dysplasia	0.1154	0.05	0.17	[25, 26]
Initial proportion of OPMDs containing moderate dysplasia	0.0440	0.03	0.06	[25, 26]
Initial proportion of OPMDs containing severe dysplasia	0.0288	0.015	0.05	[25, 26]
Probability of early stage cancer in malignant lesions	0.2731	0.5*BCE	2*BCE	[7]
Probability of presenting for visual exam in the no- screening strategy	0.30	0.15	0.45	Expert opinion
Annual probability of mild dysplasia regressing to benign lesion	0.04	0.5*BCE	2*BCE	[27]
Annual probability that mild dysplasia progresses to moderate dysplasia	0.0003	0.5*BCE	2*BCE	[28], Calibration
Annual probability of malignant transformation (MT) of moderate dysplasia	0.035	0.5*BCE	2*BCE	[28]
Annual probability of malignant transformation of low risk dysplasia^a^	0.029	0.5*BCE	2*BCE	Estimated as -15% of moderate dysplasia
Annual probability of malignant transformation of high risk dysplasia^b^	0.040	0.5*BCE	2*BCE	Estimated as +15% moderate dysplasia
Annual probability of malignant transformation of severe dysplasia	0.084	0.5*BCE	2*BCE	[28]
Annual probability of progression of early stage cancer to late stage cancer	0.046	0.5*BCE	2*BCE	[7], Calibration*
Annual probability of metastasis for untreated late stage oral cancer^‡^	0.391	0.5*BCE	2*BCE	[7], Calibration*
Annual probability of death from distant metastasis of oral cancer	0.127	0.5*BCE	2*BCE	[29]
Probability of surgical mortality	0.002	0.5*BCE	2*BCE	[30]
Probability of visual oral cavity exam for patients in non-screening strategy	0.13	0.5*BCE	2*BCE	[21, 22]
Probability of adherence to surveillance in strategies involving visual or POCOCT-based surveillance	0.92, 0.82, 0.72	0.5	1.0	[31, 32]
Probability of adhering to confirmatory biopsy recommendation after positive surveillance test	0.92	0.5	1.0	[31]
Probability of recommendation for biopsy of a lesion previously categorized as benign (by POCOCT or biopsy).	0.20	0.5*BCE	2*BCE	Expert opinion
**Test Characteristics**	**Value**	**Low**	**High**	**Source**
Sensitivity of POCOCT for differentiating benign lesions from mild dysplasia or worse (Benign|Mild)	0.90	0.86	0.92	[19]
Specificity of POCOCT for differentiating benign lesions from mild dysplasia or worse (Benign|Mild)	0.57	0.52	0.62	[19]
Sensitivity of POCOCT for differentiating benign lesions/lesions with mild dysplasia from moderate dysplasia or worse (Mild|Moderate)	0.90	0.86	0.92	[19]
Specificity of POCOCT for differentiating benign lesions/lesions with mild dysplasia from lesions with moderate dysplasia or worse (Mild|Moderate)	0.66	0.61	0.71	[19]
Sensitivity of POCOCT for differentiating lesions with low risk dysplasia from high-risk moderate dysplasia (Low|High risk)	0.91	0.87	0.93	[19]
Specificity of POCOCT for differentiating lesions with low risk dysplasia from high risk dysplasia (Low|High risk)	0.62	0.57	0.67	[19]
Sensitivity of POCOCT for differentiating lesions with benign lesions/lesions with mild or moderate dysplasia from severe dysplasia/carcinoma (Moderate|Severe)	0.92	0.89	0.94	[19]
Specificity of POCOCT for differentiating lesions with benign lesions/lesions with mild or moderate dysplasia from severe dysplasia/carcinoma (Moderate|Severe)	0.65	0.60	0.70	[19]
Sensitivity of visual exam for differentiating benign lesions from carcinoma	0.71	0.60	0.85	[33–35]
Specificity of visual exam for differentiating benign lesions from carcinoma	0.97	0.93	0.98	[33]
Sensitivity of visual exam for differentiating benign lesions from mild or moderate dysplasia	0.50	0.25	0.75	[36]
Specificity of visual exam for differentiating benign lesions from mild or moderate dysplasia	0.97	0.93	0.98	[33]
Sensitivity of scalpel biopsy for differentiating benign lesions from dysplasia or carcinoma	0.98	0.90	1	[37]
Specificity of scalpel biopsy for differentiating benign lesions from dysplasia or carcinoma	1.0	0.90	1	[37]

BCE = base case estimate

^a^This probability was applied in the diagnostic strategy where POCOCT was used to separate low- from high-risk dysplasia; low-risk entailed grouping lower degree of moderate dysplasia as well as mild dysplasia

^b^High-risk entailed the higher degree of moderate dysplasia and severe dysplasia.

^‡^Late stage disease refers to AJCC stage 3, 4a, and 4b (no distant metastasis).

We also calibrated the progression of mild to moderate dysplasia and early stage oral cancer to late stage oral cancer using similar methods; early stage represented a combination of American Joint Committee on Cancer stage I and II while the late stage combined AJCC stage III and IV (**Table 2**) according to the classification [7, 38].

**Table 2:** 5-year cancer-specific survival rates for each AJCC stage of OSCC from the SEER database for patients with age at diagnosis 60–64 years, for included oral sites from 2004–2010.

AJCC Stage of Oral Cancer	5-year cancer-specific survival	Source
Stage I	93.4%	[7]
Stage II	85.5%	[7]
Stage III	78.7%	[7]
Stage IV	66.2%	[7]

Assumptions in the base case analysis include the regression of mild dysplasia lesions to benign lesions at a rate of 30% over a 7-year period of follow up [27]. Metastatic disease rates were taken from the literature, and were calibrated to survival data by stage from Surveillance Epidemiology and End Results (SEER) data [2, 7].

### Analysis & outcomes

The model was evaluated using one million simulations and the primary outcome was life expectancy. The model was validated by comparing the life expectancy yielded by our model with life expectancy results from a recent cost-effectiveness analysis by Huang et al on the projected benefits of an oral cancer screening program in Taiwan, given the lack of trial data for visual exams in the U.S., with a predetermined expectation of falling within 5% of previously reported life expectancy [39]. The cost effectiveness analysis was based on visual screening in elevated-risk patients only (history of tobacco use) [40], and therefore we applied a hazard ratio of 1.4 for non-cancer-related mortality in our model to compare our results for an average-risk population with the most comparable group in the Huang study: former tobacco users [41, 42]. We also compared 15-year risk of oral cavity cancer for 60 year-old men with low-average risk (insignificant alcohol use, any smoking history ≤20 years) reported from a large national dataset [43]. The stability of results given changes in parameter values was evaluated in sensitivity analysis. One-way and two-way deterministic analyses were performed to assess the effects of parameter uncertainty on model results.

## Results

### Model validation

We compared our results against the published decision model of Huang et al for validation, using the authors’ population of men at age 53 years. In this patient group with diagnosed stage I OSCC, our model result was 19.17 years compared to 18.87 years reported by Huang et al, meeting the ±5% criterion as the pre-determined window of acceptability [39]. Comparison of 15-year risk of oral cavity cancer for the cohort fell within 6% of that reported by the INHANCE consortium (calculated using age, sex, risk factors in the U.S. population) [43].

### Base case analysis

The strategies involving initial referral for scalpel biopsy for all OPMDs with subsequent surgical excision of all patients with dysplasia/carcinoma and visual surveillance of negative results both yielded the highest life expectancy of 20.92 years. There was no life expectancy benefit in surgical excision for all grades of dysplasia compared with surgery for only moderate or severe dysplasia (**Table 3**). Likewise, the POCOCT strategies that entailed referral and treatment of POCOCT positive results at the benign|mild dysplasia split (with surveillance of benign results by the primary care dentist), or at the mild|moderate dysplasia split (with surveillance of mild dysplasia and benign results) resulted in the same life expectancy of 20.90 years. Therefore, there was a small difference in life expectancy with use of POCOCT to monitor mild dysplasia as compared with biopsy of all OPMDs (-0.02 years compared with prompt referral for biopsy). Other POCOCT-based strategies with higher thresholds for treatment at the low|high split or the moderate|severe dysplasia split offered lower life expectancy. Finally, the pathway modeling deferred evaluation yielded a life expectancy of at least 0.25 years less than any of the active evaluation strategies. Similar results were found for 60-year-old women (S1 Table).

**Table 3:** Comparison of life expectancy using different strategies for the base case of 60-year-old men with OPMDs.

Rank	Strategy	LE (Years)	Δ LE (Years)
1	Initial biopsy for all OPMDs, surgery for any dysplasia or malignancy, and surveillance for benign lesions	20.92	--
2	Initial biopsy for all OPMDs, surgery for moderate or severe dysplasia or carcinoma, and surveillance for mild dysplasia or benign lesions	20.92	0.00
3	POCOCT mild|moderate dysplasia diagnostic split, with surgery for moderate or severe dysplasia or carcinoma, and surveillance for benign lesions and lesions with mild dysplasia	20.90	-0.02
4	Initial biopsy for all OPMDs, surgery for any dysplasia or carcinoma and no option for surveillance	20.90	0.00
5	POCOCT benign|mild dysplasia diagnostic split, with surgery for any dysplasia or carcinoma, and surveillance for benign lesions	20.90	0.00
6	POCOCT “low risk|high risk” diagnostic split, with surgery for “higher-risk” dysplasia (encompassing more severe degree of moderate dysplasia and severe dysplasia) or carcinoma, and surveillance for benign and “lower-risk” dysplasia	20.88	-0.02
7	POCOCT moderate|severe dysplasia diagnostic split, with surgery for severe dysplasia and carcinoma, and surveillance for benign lesions or lesions with mild or moderate dysplasia.	20.87	-0.01
8	Deferral of evaluation of visible OPMDs; patients with carcinoma present clinically for treatment	20.62	-0.25

### Intermediate outcomes

The number of oral cavity carcinomas diagnosed and treated in early stage and late stage cancer were estimated in each strategy for 1 million patients **(Table 4)**. In the strategy using POCOCT with confirmatory biopsy and treatment of moderate or severe dysplasia and carcinoma, 14,102 carcinomas were diagnosed in early stage as compared to 6,819 carcinomas found in early stage with deferred evaluation. Furthermore, a total of 3,793 carcinomas arose from progression of dysplasia in this POCOCT strategy compared to the 50,290 new carcinomas that developed as the result of deferring evaluation by a specialist. In addition, there was lower incidence of metastatic disease with all POCOCT strategies compared to deferral. Compared with the hypothetical strategy of prompt specialist referral of all OPMDs, the overall number of biopsies was reduced 27% over the population lifetime (from 946 to 689 per 1000 patients) in the POCOCT strategy that referred patients with POCOCT + results at the mild|moderate split to specialists, including 29% fewer biopsies for benign lesions over the same period (721 vs. 513 per 1000 patients).

**Table 4:** Numbers of carcinomas over remaining lifetime in one million simulated 60-year-old men with OPMDs (using base case estimates for model parameters).

Strategies in order of ranking results	Numbers of Simulated Patients with Diagnoses			
	Early-stage cancer^a^	Late-stage cancer^b^	Dysplasia progressed to cancers	New cases of metastasis
Initial biopsy for all OPMDs, surgery for any dysplasia or carcinoma, and surveillance for benign lesions	13790	34959	1477	899
Initial biopsy for all OPMDs, surgery for moderate or severe dysplasia or carcinoma, and surveillance for mild dysplasia or benign lesions	13790	34959	1500	978
POCOCT mild|moderate dysplasia diagnostic split, with surgery for moderate or severe dysplasia or carcinoma, and surveillance for benign lesions and lesions with mild dysplasia	14102	32642	3793	1196
Initial biopsy for all OPMDs, surgery for any dysplasia or carcinoma	12660	33098	5316	4460
POCOCT benign|mild dysplasia diagnostic split, with surgery for any dysplasia or carcinoma, and surveillance for benign lesions	14158	32633	3774	1182
POCOCT “low risk|high risk” diagnostic split, with surgery for “higher-risk” dysplasia or carcinoma, and surveillance for benign and “lower-risk” dysplasia	16294	32824	6144	10717
POCOCT moderate|severe dysplasia diagnostic split, with surgery for severe dysplasia and carcinoma, and surveillance for benign lesions or lesions with mild or moderate dysplasia.	16645	32892	10562	22825
Deferral of evaluation of visible OPMDs; patients with carcinoma present clinically for treatment	6819	7151	50290	44575

^a^Total number of cancers diagnosed with biopsy and surgically treated in early stage (AJCC 1, 2).

^b^Total number of cancers diagnosed with biopsy and surgically treated in late stage (AJCC 3, 4a, 4b).

### Sensitivity analysis

The uncertainty around key model inputs was examined using a sensitivity analysis. Within the tested ranges of variable values, model results were most sensitive to the probability of adherence to surveillance protocols, adherence to biopsy recommendations, probability of death from metastatic OSCC and the probability of developing distant metastasis with untreated OSCC. When the monthly probability of metastasis from untreated OSCC was less than 0.01, the most favorable strategy was the use of POCOCT to refer only those with a POCOCT + test at the mild|moderate split. In two-way sensitivity analysis, the adherence to surveillance and to biopsy recommendations were varied and favored the use of POCOCT to refer and treat only those with a POCOCT + test at the mild|moderate split when adherence to both was ≥0.88.

## Discussion

Because late-stage diagnosis of OSCC has been a barrier to improving cancer-specific outcomes, emphasis has been placed on the need for more effective screening and specifically, adjunctive diagnostic testing to aid OPMD characterization and management. The advantage of a cytopathological test is that lesions can be retested at follow-up visits, thereby allowing the monitoring of evolving OPMDs in a primary dental setting. We built a decision-analytic model based on a diagnostic accuracy trial of a point-of-care test for precision OPMD characterization and compared the effectiveness of this risk-assessment tool with common clinical strategies [25]. A key finding about the management of mild dysplasia was that our model indicated no benefit for surgical treatment instead of monitoring for malignant transformation. Second, we found applying POCOCT with a mild|moderate split would likely provide similar life expectancy (7 days difference) for 60-year-old men and women with OPMDs compared with a more conservative hypothetical approach of routine referral to a specialist for scalpel biopsy. To provide context, the comparative advantage with use of routine biopsy is comparable to population-level life expectancy benefits of other diagnostic tests or interventions in other clinical scenarios (e.g. breast tomosynthesis, renal mass biopsy) that are widely utilized [44–46]. However, these are situations that reasonably entail a preference-based choice due to added inconvenience to patients. Our findings also reflect assumptions of limited patient adherence to POCOCT surveillance protocols. Thus, POCOCT and biopsy-based diagnostic evaluation may be weighed using patient preferences, considering the invasive nature and inconvenience of referral for biopsy.

Furthermore, when adherence to surveillance was higher (probabilities ≥0.88), POCOCT-based management for OPMDs extended life expectancy compared with referral, due to the possibility of surgical mortality (i.e. overtreatment, where harms of surgery outweighed risks of missing malignant transformation). This further supports the potential of risk-based OPMD management using this point-of-care test in the primary dental clinics, since patients could elect through informed decisions to serially monitor visible lesions non-invasively and receive call-backs if surveillance visits were missed. The model results were also driven by the prevalence of severely dysplastic or malignant OPMDs at the time of initial detection. Although POCOCT’s imperfect sensitivity meant that more dysplasia or cancers were initially missed than with initial biopsy, repeat visits for surveillance served to capture early-stage OSCC before progression to late-stage cancer, substantially lowering the rates of metastatic disease development compared with deferred evaluation.

In 2014, the United States Preventive Services Task Force concluded that there was insufficient evidence to recommend routine screening for oral cavity cancer by primary care physicians even for high-risk patients [47]. However, oral examinations are routinely performed by dentists, and therefore opportunistic screening for oral cancer by dentists is recommended [10]. More recent patient data supports the efficacy of screening and the cost effectiveness of screening programs in high-risk populations specifically [40, 47, 48]. Dedhia et al recommended a community-based oral cancer screening program for high-risk individuals above 40 years of age in the United States in their decision analysis informed by screening data from India [49]. Based on clinical trial results, Subramanian et al also found visual-exam based screening efforts to be most cost-effective in high-risk patients in India [48]. These studies emphasized the impact on population life expectancy of early stage detection of oral cancer, and with visual inspections beginning in middle age. Screening effectiveness in moderate risk groups has historically been hampered by the lower positive predictive value of visual-tactile exam. Based on our analysis, further evaluation of POCOCT is warranted to re-explore the benefit and harms of broadening oral cancer screening in primary care, as well as patient and clinician preferences and acceptability.

The primary limitations of our study include the simplifying assumptions inherent to any decision analytic model. Retrospective studies on the progression of dysplastic lesions may not reflect accurately the natural progression of dysplasia to carcinoma, though we estimated the likely transformation rates using calibration targets from patient studies [43]. We addressed this limitation and other parameter uncertainty through extensive sensitivity analysis, through a range of estimates of progression rates as well as model validation. The POCOCT will require further clinical testing for test performance in the primary care setting. The sensitivity and specificity at each diagnostic threshold were varied across the reported 95% confidence intervals with no difference in comparative life expectancy rankings. We also assumed surveillance lasting up to five years, and that lesions not meeting treatment criteria in those strategies would remain highly indolent at the end of the surveillance period with no further risk of progressing to metastasis. It is plausible that a small proportion of lesions might transform over longer periods. Finally, it is possible that referral to oral medicine specialists might result in observation of lesions without biopsy when there are no visual features suspicious for high-grade dysplasia or malignancy. It is not clear in the literature how often this practice would occur, and life expectancy may be approximated using strategies of monitoring mild dysplasia, but the tradeoffs are not explicitly modeled. Further, comparative costs and quality-of-life considerations are not measured in our outcomes as would be in a cost-effectiveness analysis.

In conclusion, we assessed the projected long-term clinical outcomes of point-of-care characterization of OPMDs with a brush-based, cytological artificial intelligence-assisted device. Using POCOCT, a surveillance protocol for POCOCT negative results (both benign|mild and mild|moderate dysplasia splits) in visible lesions extended life expectancy compared to deferred evaluation of the oral cavity for OPMDs. Even with suboptimal patient adherence, POCOCT offered only slightly less life expectancy benefit than routine specialist referral for biopsy while substantially reducing referrals and biopsies for benign lesions or mild dysplasia. The major potential risk of a surveillance program using POCOCT is the possibility for a false negative result, particularly in patients who do not adhere to surveillance and have limited opportunity for retesting. One solution may be to offer patients bifurcated options based on their preferences for non-invasive monitoring versus immediate intervention. These results may guide design of clinical validation trials that establish the benefit-risk profiles of more tailored management, including benefits of more frequent detection of early-stage OSCC and high-grade dysplasia, and substantially fewer specialist referrals for low-risk lesions.

## Supporting information

S1 Table Life expectancy results for 60-year-old women with PMOLs. (DOCX)
