# Supplementary material for: Point-of-care characterization and risk-based management of oral lesions in primary dental clinics: A simulation model
Source: PLoS One. 2020 Dec 31;15(12):e0244446. doi: 10.1371/journal.pone.0244446 (PMC7774939; doi:10.1371/journal.pone.0244446)
Supplement: S1 Table — (DOCX) [file pone.0244446.s001.docx]

**Supplemental Table 1**: Life expectancy results for 60-year-old women with PMOLs.

| **Rank** | **Strategy** | **LE (Years)** | **Δ LE (Years)** |
| --- | --- | --- | --- |
| 1 | Initial biopsy for all PMOLs, surgery for any dysplasia or malignancy, and surveillance for benign lesions | 22.32 | -- |
| 2 | Initial biopsy for all PMOLs, surgery for moderate or severe dysplasia or malignancy, and surveillance for mild dysplasia or benign lesions | 22.31 | -0.01 |
| 3 | POCOCT characterization with surgery for moderate or severe dysplasia or malignancy, and otherwise surveillance | 22.30 | -0.01 |
| 4 | Initial biopsy for all PMOLs, surgery for any dysplasia or malignancy and no option for surveillance | 22.30 | 0.00 |
| 5 | POCOCT characterization with surgery for any dysplasia or malignancy and surveillance for benign lesions | 22.29 | -0.01 |
| 6 | POCOCT characterization with surgery for high-risk moderate dysplasia, severe dysplasia and malignancy, and otherwise surveillance | 22.28 | -0.01 |
| 7 | POCOCT characterization with surgery for severe dysplasia and malignancy, and otherwise surveillance | 22.27 | -0.01 |
| 8 | No testing, cancerous lesions present clinically for treatment | 21.98 | -0.29 |
